# Running-induced neurogenesis reduces CA1 perineuronal net density without substantial temporal delay

**DOI:** 10.1186/s13041-024-01138-x

**Published:** 2024-09-02

**Authors:** Dylan J. Terstege, Duneesha Goonetilleke, Cindy K. Barha, Jonathan R. Epp

**Affiliations:** 1 Department of Cell Biology and Anatomy, Cumming School of Medicine, University of Calgary, HMRB 162, Health Sciences Centre, 3330 Hospital Drive NW, Calgary, AB T2N 4N1 Canada; 2 Hotchkiss Brain Institute, University of Calgary, 3330 Hospital Drive NW, Calgary, AB T2N 4N1 Canada; 3 Department of Kinesiology, University of Calgary, 376 Collegiate Blvd NW, Calgary, AB T2N 4V8 Canada

**Keywords:** Cell proliferation, Aerobic exercise, Extracellular matrix

## Abstract

Aerobic exercise has many effects on brain function, particularly at the hippocampus. Exercise has been shown to increase the rate of adult neurogenesis within the dentate gyrus and decrease the density of perineuronal nets in area CA1. The relationship between the rate of neurogenesis and the density of perineuronal nets in CA1 is robust; however, these studies only ever examined these effects across longer time scales, with running manipulations of 4 weeks or longer. With such long periods of manipulation, the precise temporal nature of the relationship between running-induced neurogenesis and reduced perineuronal net density in CA1 is unknown. Here, we provided male and female mice with home cage access to running wheels for 0, 1, 2, or 4 weeks and quantified hippocampal neurogenesis and CA1 perineuronal net density. In doing so, we observed a 2-week delay period prior to the increase in neurogenesis, which coincided with the same delay prior to decreased CA1 perineuronal net density. These results highlight the closely linked temporal relationship between running-induced neurogenesis and decreased perineuronal net expression in CA1.

**Supplementary Information:**

The online version contains supplementary material available at 10.1186/s13041-024-01138-x.

## Background

Aerobic exercise has been shown to modulate short term cognitive performance and long term cognitive outcomes in many ways [1, 2]. In particular, running has been found to impact learning and memory ability in a timing-dependent manner. Running prior to learning aids in the formation of new memories [3–5]. However, running after learning promotes the forgetting of recently acquired information [6–9]. One of the mechanisms which seems to contribute to this relationship between running and cognition is adult hippocampal neurogenesis, which increases with running [10, 11]. With increased neurogenesis, the excitability of the dentate gyrus changes, which has knock-on effects across the hippocampus.

Among these effects of increased neurogenesis is a decrease in the density of perineuronal nets (PNNs) in CA1 [9]. PNNs are an extracellular matrix structure which regulate the excitability and potential for plasticity of the cells that they surround. As such, they have been associated with maintaining stability in synaptic connections underlying learning and memory processes [12, 13]. With increased hippocampal neurogenesis and consequent decreases in CA1 PNN density, it has been proposed that this increase in potential for CA1 plasticity may contribute to both promoting the acquisition of new memories and the destabilization of previously acquired memories [9, 14].

While the relationship between neurogenesis and CA1 PNN density is robust, the temporal nature of this relationship is unknown. In the present study, we investigate voluntary running protocols of different durations to assess whether increased expression density of neurogenesis markers precedes decreased expression density of PNNs.

## Methods

We examined brains from male and female 12-week-old C57BL6/J mice (Jackson Labs) following 0, 1, 2, or 4 weeks of running wheel access. Wireless low-profile running wheels were presented to mice in the home cage (*n* = 5 mice/cage) for the duration of the manipulation, only being removed to change batteries and to offload data every second day [15].

When running wheels were removed at the end of the manipulation, mice were injected intraperitoneally with 2’-deoxy-5-ethynyluridine (EdU; NE08701; BioSynth) at 0.1 mg/g. EdU is a nucleoside analog to thymidine and is incorporated into dividing cells during DNA synthesis. To ensure that we were only labelling actively dividing cells, mice were deeply anesthetized 24 h later using isofluorane and transcardially perfused with 20 mL of 0.1 M phosphate-buffered saline (PBS) followed by 20 mL of 4% formaldehyde. Brains were postfixed in 4% formaldehyde for 24 h and then transferred to 30% sucrose solution prior to sectioning on a Cryostat (Leica CM1950) in twelve-series at a thickness of 50 μm. Sectioned tissue was stored in antifreeze at -20 C.

To label EdU, tissue was washed (3 × 10 min) in 0.1 M Tris-buffered saline (TBS) and then incubated in the dark for 30 min in freshly prepared solution of 40 mM CuSO4, 2 mM Sulfo-Cy3 azide, and 1 M sodium ascorbate. Following incubation, tissue was washed in 0.1 M TBS. PNNs were labelled using a 1:100 dilution of WFA-TRITC (R-3101-1; EY Laboratories) and 0.02% tween-20 in carbo-free blocking buffer (VectorLABS). All tissues were stained with DAPI (1:1000 in PBS; 30 min), mounted to plain glass slides, and cover slipped with PVA-DABCO mounting medium.

Quantification was performed on an Olympus BX63 fluorescent microscope. EdU-labelled cells were quantified manually at 60X magnification throughout the granule cell layer of the dentate gyrus. PNNs were imaged at 10X magnification and counted manually across the entire hippocampus. Labels were only quantified within the granule cell layers and all counts were normalized by region area, which was traced in *ImageJ* using DAPI labelling as reference.

## Results

**Fig. 1:**
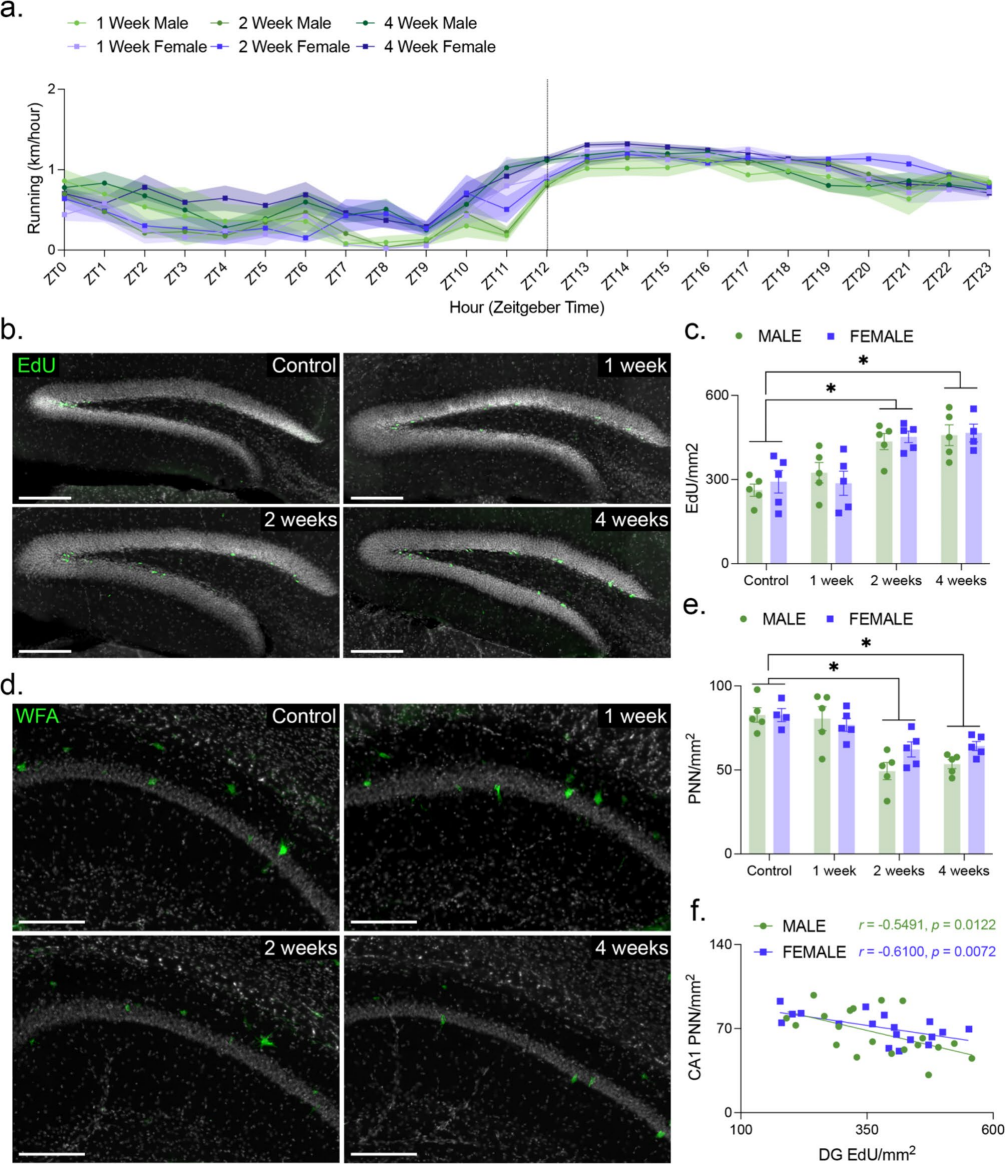
Changes in neurogenesis associated with running are closely linked temporally to changes in PNN density in CA1. (**a**) Mean hourly running distance over the course of the day. Lights were turned on at ZT0 and turned off at ZT12. (**b**) Representative images of EdU^+^ cells in the subgranular zone of the dentate gyrus. Scale bar represents 100 μm. (**c**) The influence of sex and duration of running wheel access on the density of EdU^+^ cells. The density of EdU^+^ cells in the dentate gyrus increased with running (Two-Factor ANOVA, Effect of Running; F_3,31_ = 15.71, *P* < 0.0001). Subsequent Šídák *post hoc* analyses revealed that this effect was being driven by 2-week (control vs. 2 weeks, *P* = 0.001) and 4-week (control vs. 4 weeks, *P* < 0.001) groups. At each running duration, the Cohen’s *d* effect sizes between males and females were as follows: control = 0.415, 1 week = -0.42, 2 weeks = 0.298, and 4 weeks = 0.103. (**d**) Representative images of PNNs in CA1. Scale bar represents 100 μm. (**e**) The influence of sex and duration of running wheel access on the density of PNNs. The density of PNNs in CA1 decreased with running (Two-Factor ANOVA, Effect of Running; F_3,31_ = 17.97, *P* < 0.0001). Subsequent Šídák *post hoc* analyses revealed that this effect was being driven by 2-week (control vs. 2 weeks, *P* < 0.0001) and 4-week (control vs. 4 weeks, *P* < 0.0001) groups. At each running duration, the Cohen’s *d* effect sizes between males and females were as follows: control = 0.00268, 1 week = -0.288, 2 weeks = -1.19, and 4 weeks = -1.79. (**f**) EdU density and CA1 PNN density for each animal examined in this study. In both males (y = -0.09817x + 102.8; *r* = -0.5491, *P* = 0.0122) and females (y = -0.06209x + 94.43; *r* = -0.6100, *P* = 0.0072), the density of PNNs in CA1 was anticorrelated with the density of EdU^+^ cells in the dentate gyrus. Data are mean ± SEM. *P < 0.05

Mice showed typical patterns of daily running, with the majority of wheel running taking place during the dark phase of the light/dark cycle (Fig. 1a). Furthermore, we observed a significant main effect of running protocol duration on the density of EdU-labelled cells in the dentate gyrus (Fig. 1c). Šídák’s multiple comparison revealed no significant differences in the density of EdU-labelled cells between 0- and 1-week groups. By 2- and 4-weeks, the rate of cell proliferation is significantly higher than the 0-week control group. There was no main effect of sex on the density of EdU-labelled cells.

As the rate of cell proliferation does not increase immediately upon starting to run, we predicted that the rate of PNN degradation in CA1 might also be delayed. Our results confirm this prediction (Fig. 1e). While the density of PNNs did not change in the dentate gyrus and CA3, there was a significant main effect of running on the density of these extracellular matrix structures in CA1. Similar to the cell proliferation results, Šídák’s multiple comparison revealed no significant differences in the density of PNNs between 0- and 1-week groups. By 2- and 4-weeks, the density of PNN labelling in CA1 is significantly lower than the 0-week control group. Furthermore, the density of PNN labelling in CA1 was found to be anti-correlated with the density of EdU-labelled cells in the dentate gyrus (Fig. 1f). There was no main effect of sex on the regional densities of PNN labelling; however, with 2 and 4 weeks of running wheel access the decrease in CA1 PNN was found to be more pronounced in males than in females.

Prior research has demonstrated that conditions which increase the rate of neurogenesis, such as voluntary running or pharmacological treatment with memantine, decrease the density of CA1 PNNs [9]. Conditions which decrease neurogenesis, such as natural aging or pharmacological treatment with temozolomide, have been shown to increase the expression density of PNNs in CA1 [9]. Furthermore, optogenetic stimulation of adult-born neurons in the DG, thus mimicking the alterations in DG activity which are observed with increased neurogenesis, is sufficient to decrease the density of CA1 PNNs without altering the rate of neurogenesis [9]. This suggests that changes in neurogenesis likely precede decreased PNN density at CA1; however, the temporal nature of this relationship had not been studied at any gradient finer than 4 weeks. Here, we demonstrate that both changes occur within the second week of running wheel access and follow similar trajectories. These results highlight the closely linked temporal relationship between running-induced neurogenesis and decreased PNN expression in CA1.

### Electronic supplementary material

Below is the link to the electronic supplementary material.

Supplementary Material 1

## Data Availability

The data that support the findings of this study are available in the manuscript and available from the corresponding author upon reasonable request.

## Abbreviations

EdU: 2’-deoxy-5-ethynyluridine
PBS: Phosphate-buffered saline
PNN: Perineuronal net
TBS: Tris-buffered saline
WFA: Wisteria floribunda lectin
